# Supplementary material for: Systematic review and meta-analysis of ex-post evaluations on the effectiveness of carbon pricing
Source: Nat Commun. 2024 May 16;15:4147. doi: 10.1038/s41467-024-48512-w (PMC11099057; doi:10.1038/s41467-024-48512-w)
Supplement: Supplementary file 1 — Supplementary Information [file 41467_2024_48512_MOESM1_ESM.pdf]

Effectiveness of carbon pricing – A systematic review and meta-analysis of the ex-post literature

Supplementary Information

Table S1: Average treatment effects, estimated with a variety of models

	Full sample	Full sample with outliers	Full sample clustered	Full sample, PET	Full sample, PEESE	Full sample, fixed effects (clustered)	Subsample: low RoB	Subsample: adequately powered	Subsample: low RoB and adequately powered	Subsample: without Chinese pilot ETS
Average treatment effect	-10.42 (0.76)	-12.52 (1.20)	-10.42 (0.76)	-7.31 (1.37)	-12.50 (1.20)	-4.39 (0.59)	-10.8 (1.03)	-6.83 (0.61)	-6.35 (0.70)	-8.14 (0.87)
SE				-0.97 (0.14)						
SE^2					0.00 (0.00)					

Note: The standard errors are displayed in parentheses.

Table S2: Average treatment effects for individual carbon pricing schemes

	Full sample	Low risk of bias	Adequately powered estimates	Low risk of bias and adequately powered
Chinese pilot ETS	-13.14	-12.93	-8.66	-8.35
	(1.04)	(1.29)	(1.24)	(1.33)
EU ETS	-7.27	-7.43	-7.79	-6.17
	(1.66)	(3.26)	(1.38)	(2.18)
BC carbon tax	-5.43	-5.32	-5.66	-5.55
	(2.15)	(2.36)	(1.41)	(1.42)
RGGI	-21.05	-21.02	-6.10	-6.10
	(3.63)	(3.74)	(5.21)	(4.65)
cross-country	-5.85	-4.93	-4.25	-4.65
	(3.04)	(4.27)	(2.10)	(2.36)
Tokyo ETS	-7.29	-5.97	-5.46	-5.77
	(3.29)	(5.96)	(2.32)	(3.21)
UK carbon price support	-9.78	-7.81	-8.49	-7.02
	(3.44)	(4.89)	(2.43)	(3.36)
Saitama ETS	-6.30	-1.06	-5.27	-1.06
	(3.51)	(5.96)	(2.14)	(3.21)
California CaT	-18.90		-15.10	
	(5.19)		(5.21)	
Finnish carbon tax	-14.83	-16.59		
	(5.21)	(8.58)		
Korea ETS	-3.16		-1.58	
	(6.34)		(5.17)	
Quebec ETS	-8.54	-10.62	-7.92	-9.38
	(4.03)	(6.16)	(2.95)	(3.77)
Swedish carbon tax	-10.17	-5.43		
	(4.06)	(6.75)		
Australian carbon tax	0.16		0.17	
	(5.83)		(3.90)	
Swiss ETS	14.25			
	(12.49)			

Note: The standard errors are displayed in parentheses.

Heterogeneity assessment

The heterogeneity assessment conducted using Bayesian model averaging includes the following explanatory variables.
Carbon pricing scheme Dummy variables are included for *BC_carbon_tax*, *Chinese_pilot_ETS*, *Finnish_carbon_tax*, *Quebec_ETS*, *RGGI*, *Saitama_ETS*, *Swedish_carbon_tax*, *Swiss_ETS*, *Tokyo_ETS*. The EU ETS together with the UK carbon price support are used as a benchmark. The remaining schemes are collected by *other_schemes*. This variable also includes cross-country studies.
Carbon price level We include a variable (*log_carbon_price*) for the mean carbon price level during the period assessed in the respective study. If the primary study reports the carbon prices of the analysed carbon tax or emissions trading scheme, we use these prices. If this information is not available in the study, we use external information from two sources. Our preferred source is the carbon

price data provided by the World Bank Carbon Pricing Dashboard, which we downloaded on July 1, 2022. For the EU ETS data on carbon prices are not provided by the World Bank. We instead use data provided by the International Carbon Action Partnership (ICAP) which we downloaded on February 1, 2022. The prices are averaged across the assessment period for each effect size. We fill missing values in the carbon pricing data with the nearest available carbon price in the time series. If the effect of multiple carbon pricing schemes was analyzed together, we compute a weighted average, where the weights are the relative size of the emissions covered by each scheme. All price levels are converted to US$ using exchange rates provided by the International Monetary Fund (IMF) and sourced via the World Bank data API with help of the WDI R package. Finally, all prices are deflated to constant 2010 US$. Inflation data is again provided by the IMF and sourced as described above. The mean prices are logarithmised in our BMA.

Sector coverage For the assessment we distinguish between the *industrial_sectors* energy and industry, on the one hand, and the transport and housing sectors, on the other hand. The latter are used as the benchmark in the regressions. If a study is conducted across multiple emitting sectors, the variable is coded for the general coverage of the carbon pricing scheme. Commonly the assessed carbon pricing schemes are restricted to either the energy and industry sectors or to the transport and housing sectors. In some cases, when mainly the former sectors are covered and a small fraction of the latter, the scheme is considered to cover the *industrial_sectors*.

Policy design The dummy variable *tax* captures all carbon pricing schemes that are implemented as carbon taxes, while the benchmark are cap-and-trade schemes.

Study period The variable *duration* captures the time between the introduction of the assessed policy and the last included observation.

Study design Motivated by the observation by Green (2021) that synthetic control designs find the largest emission reductions, we test for the relevance of this quasi-experimental method in our comprehensive sample of studies (*synthetic_control*). We find that the method can explain some of the heterogeneity, but that it is associated with lower emission reduction effects, compared to other study designs.

Data granularity We capture whether the data studied in the primary assessments was on the regional, city, sector, firm, plant, or airline level (*Data_Region*, *Data_City*, *Data_Sector*, *Data_Firm*, *Data_Plant*, *Data_Airline*). The benchmark is the country level. We also capture, whether the study uses yearly (*Data_Year*), monthly (*Data_Month*) or more granular data. The benchmark is more granular data. Testing for the spatial and temporal granularity of the data suggests that only the use of city level data compared to the country level explains some of the heterogeneity in reported effect sizes. The coefficient of the variable is positive and has a high PIP of 0.78. This indicates that studies that focus on a narrow area perhaps do not account for wider effects of carbon pricing and therefore report smaller effect sizes.

Fuel coverage If the study assesses only one fuel type, we capture which fuel

type is studied (*Gasoline*, *Gas*, *Coal*). The benchmark are studies across all fuels. These are not suggested by the BMA to be relevant to explain the effect size heterogeneity.

Data transformations We capture whether the study assesses the emissions in the dependent variable as the total (*DVTotal*), compared to the benchmark of per capita emissions, and whether it uses a level-level regression specification (*TransLevelLevel*), compared to a log-level specification. Both are not suggested by the BMA to be relevant to explain the effect size heterogeneity.

Bias To capture the biases in study designs and publication bias, assessed in our study, we include the standard error of the transformed effect sizes (*SE_percent*) and a dummy variable for all studies with low risk of bias (*Less_Bias*).

Table S3: BMA with alternative priors

	Uniform g-prior				Dilution prior				BRIC g-prior			
	PIP	Post Mean	Post SD	Sign	PIP	Post Mean	Post SD	Sign	PIP	Post Mean	Post SD	Sign
RGGI	0.9995	-28.4470	5.0888	0.0000	1.0000	-30.0563	4.6098	0.0000	1.0000	-30.2213	4.5909	0.0000
Chinese_pilot_ETS	0.9912	-9.7614	2.2285	0.0000	0.9989	-9.6434	1.6511	0.0000	0.9992	-9.6070	1.5812	0.0000
Swiss_ETS	0.8043	14.3507	8.9349	1.0000	0.3463	5.9242	8.8821	1.0000	0.2572	4.3926	8.0679	1.0000
Data_City	0.7759	11.3897	7.6322	1.0000	0.3126	4.2109	6.8207	1.0000	0.2214	2.9425	5.9712	1.0000
duration	0.7614	-0.6351	0.4625	0.0000	0.4002	-0.3015	0.4064	0.0000	0.3519	-0.2731	0.4002	0.0000
synthetic_control	0.4180	2.8697	3.8671	0.9971	0.0385	0.1941	1.1771	0.9282	0.0182	0.0768	0.7402	0.8644
tax	0.4095	-3.1126	4.2424	0.0054	0.1592	-1.4357	3.5543	0.0021	0.1164	-1.0826	3.1861	0.0009
BC_carbon_tax	0.3834	3.8998	5.6509	0.9997	0.1526	1.6532	4.2367	1.0000	0.1113	1.2176	3.7064	1.0000
Swedish_carbon_tax	0.3627	-3.0514	4.6512	0.0171	0.6224	-6.2157	5.2536	0.0002	0.6623	-6.7519	5.2044	0.0001
Coal	0.3250	-2.5837	4.2609	0.0000	0.1232	-1.0401	3.0554	0.0000	0.0865	-0.7370	2.6248	0.0000
Less_Bias	0.3017	1.1583	2.0038	1.0000	0.0559	0.1938	0.9038	1.0000	0.0306	0.1002	0.6485	1.0000
Finnish_carbon_tax	0.2509	-2.8870	5.6951	0.0004	0.1636	-2.0801	5.1188	0.0000	0.1335	-1.7199	4.7358	0.0000
TransLevelLevel	0.1890	-0.6987	1.6730	0.0000	0.0405	-0.1276	0.7251	0.0000	0.0252	-0.0761	0.5564	0.0000
Data_Region	0.1222	-0.3978	1.2963	0.0143	0.0470	-0.1673	0.8779	0.0018	0.0319	-0.1117	0.7179	0.0002
log_carbon_price	0.0880	0.1491	0.6242	0.9883	0.0155	0.0192	0.2230	0.9142	0.0106	0.0127	0.1819	0.9030
Data_Sector	0.0853	0.2481	1.0243	0.9851	0.0206	0.0578	0.5007	0.9925	0.0133	0.0362	0.3954	0.9937
Gas	0.0797	-0.4374	1.8843	0.0000	0.0154	-0.0672	0.7297	0.0000	0.0100	-0.0421	0.5760	0.0000
other_schemes	0.0471	-0.3090	2.0004	0.0000	0.0148	-0.1072	1.1901	0.0000	0.0098	-0.0721	0.9781	0.0000
Tokyo_ETS	0.0431	0.1459	1.0331	0.9966	0.0112	0.0353	0.5134	1.0000	0.0071	0.0219	0.4057	1.0000
industrial_sectors	0.0429	-0.0447	0.7686	0.3686	0.0106	0.0015	0.3050	0.5888	0.0071	0.0038	0.2327	0.6747
Data_Firm	0.0418	0.0709	0.5352	0.9717	0.0122	0.0221	0.2952	0.9876	0.0083	0.0153	0.2445	0.9909
Data_Plant	0.0341	0.0375	0.4701	0.8218	0.0106	0.0163	0.2736	0.9381	0.0074	0.0118	0.2284	0.9595
DVTotal	0.0340	0.0349	0.5058	0.7291	0.0084	0.0043	0.2076	0.6427	0.0062	0.0039	0.1796	0.6788
Saitama_ETS	0.0314	0.0542	0.6530	0.9556	0.0097	0.0193	0.3682	0.9961	0.0064	0.0131	0.2995	1.0000
SE_percent	0.0300	-0.0001	0.0017	0.1833	0.0083	0.0000	0.0009	0.6274	0.0055	0.0000	0.0007	0.7453
Gasoline	0.0300	-0.0158	0.6242	0.4134	0.0083	0.0018	0.2797	0.6259	0.0054	0.0020	0.2210	0.6983
Quebec_ETS	0.0297	-0.0624	0.8224	0.0233	0.0089	-0.0195	0.4548	0.0001	0.0057	-0.0120	0.3608	0.0001
Data_Month	0.0286	-0.0187	0.5163	0.2463	0.0081	-0.0062	0.2653	0.1974	0.0057	-0.0045	0.2209	0.1965
Data_Year	0.0286	0.0105	0.4140	0.6746	0.0081	0.0054	0.2161	0.7795	0.0056	0.0041	0.1794	0.8222
Data_Airline	0.0276	-0.0025	0.7496	0.4954	0.0073	0.0016	0.3707	0.6884	0.0049	0.0017	0.3030	0.8105
(Intercept)	1.0000	-5.9894			1.0000	-6.4805			1.0000	-6.5557		

Note: For each of the model specifications and each variable, the table provides the posterior inclusion probability (PIP), the mean (Post Mean) and standard deviation (Post SD) of the posterior distribution for a respective explanatory variable and the share of the meta-regressions where the variable is estimated with a positive sign (Sign). The variables are ordered by their PIP in the first model, which is the main model also presented in the main text.

Table S4: BMA without dummy variables per scheme

	PIP	Post Mean	Post SD	Cond.Pos.Sign
log_carbon_price	1.0000	3.8256	0.6633	1.0000
duration	1.0000	-1.5770	0.2594	0.0000
TransLevelLevel	0.9994	-7.1611	1.5894	0.0000
synthetic_control	0.9909	10.2756	2.8058	1.0000
Data_City	0.9618	16.6968	5.8521	1.0000
Coal	0.8677	-10.0338	5.2010	0.0000
Gasoline	0.1481	0.7873	2.3816	0.9999
Data_Airline	0.1382	0.9498	2.8731	1.0000
Data_Plant	0.1233	0.3781	1.2462	1.0000
industrial_sectors	0.1131	-0.3152	1.1226	0.0054
Gas	0.0947	-0.4457	1.7834	0.0000
Data_Sector	0.0868	0.2182	0.9397	1.0000
DVTotal	0.0790	-0.1766	0.8517	0.0022
Data_Month	0.0749	-0.2994	1.6795	0.0326
Less_Bias	0.0661	0.0967	0.5369	0.9989
Data_Firm	0.0621	-0.1098	0.6638	0.0082
Data_Year	0.0572	-0.0917	0.7732	0.0901
tax	0.0473	-0.0060	0.5018	0.6558
Data_Region	0.0450	-0.0007	0.3578	0.6033
SE_percent	0.0432	-0.0000	0.0019	0.0508
(Intercept)	1.0000	-14.3036		

Note: The table provides for each variable the posterior inclusion probability (PIP), the mean (Post Mean) and standard deviation (Post SD) of the posterior distribution for a respective explanatory variable and the share of the meta-regressions where the variable is estimated with a positive sign (Sign). The variables are ordered by their PIP in the first model, which is the main model also presented in the main text.

Table S5: Studies included in the systematic review

Authors (year)	Title	Scheme	Studied sector	Study design
Leslie (2018)	Tax induced emissions? Estimating short-run emission impacts from carbon taxation under different market structures	Australian carbon tax	Energy	control/treatment
Lawley & Thivierge (2018)	Refining the Evidence: British Columbia's Carbon Tax and Household Gasoline Consumption	BC carbon tax	Transport	price elasticity
Metcalf (2019)	On the Economics of a Carbon Tax f or the United States	BC carbon tax	Whole economy	DiD

Table S5 – *Continued from previous page*

Authors (year)	Title	Scheme	Studied sector	Study design
Pretis (2022)	Does a Carbon Tax Reduce CO2 Emissions? Evidence From British Columbia	BC carbon tax	All sectors covered by the carbon price	DiD
Rivers & Schaufele (2015)	Salience of carbon taxes in the gasoline market	BC carbon tax	Transport	price elasticity
Xiang & Lawley (2019)	The impact of British Columbia's carbon tax on residential natural gas consumption	BC carbon tax	Buildings	DiD, price elasticity
Erutku & Hildebrand (2018)	Carbon Tax at the Pump in British Columbia and Quebec	BC carbon tax, Quebec ETS	Transport	price elasticity
Bartram et al. (2022)	Real effects of climate policy: Financial constraints and spillovers	California CaT	Industry	DiD
Martin & Saikawa (2017)	Effectiveness of state climate and energy policies in reducing power-sector CO2 emissions	California CaT, RGGI	Energy	DiD
Cao et al. (2021)	When carbon emission trading meets a regulated industry: Evidence from the electricity sector of China	Chinese pilot ETS	Energy	DiD
Chen et al. (2020)	Carbon emission curbing effects and influencing mechanisms of China's Emission Trading Scheme: The mediating roles of technique effect, composition effect and allocation effect	Chinese pilot ETS	Whole economy	DiD
Cui et al. (2021)	The effectiveness of China's regional carbon market pilots in reducing firm emissions	Chinese pilot ETS	All sectors covered by the carbon price	DiD
Dong et al. (2019)	Can a carbon emission trading scheme generate the Porter effect? Evidence from pilot areas in China	Chinese pilot ETS	Whole economy	DiD
Dong et al. (2020)	The validity of carbon emission trading policies: Evidence from a quasi-natural experiment in China	Chinese pilot ETS	All sectors covered by the carbon price	DiD
Gao et al. (2020)	Evaluation of effectiveness of China's carbon emissions trading scheme in carbon mitigation	Chinese pilot ETS	All sectors covered by the carbon price	DiD
Hu et al. (2020)	Can carbon emission trading scheme achieve energy conservation and emission reduction? Evidence from the industrial sector in China	Chinese pilot ETS	All sectors covered by the carbon price	DiD
Li, Shu & Jin (2021)	Environmental regulation, carbon emissions and green total factor productivity: a case study of China	Chinese pilot ETS	All sectors covered by the carbon price	DiD
Lixiang & Chuxiao (2020)	Research on Fossil Fuel Related Carbon Emissions Reduction Scheme Effects	Chinese pilot ETS	All sectors covered by the carbon price	DiD

Table S5 – *Continued from previous page*

Authors (year)	Title	Scheme	Studied sector	Study design
Ma et al. (2022)	Can China's carbon emissions trading scheme achieve a double dividend?	Chinese pilot ETS	All sectors covered by the carbon price	DiD
Ouyang et al. (2020)	Factors behind CO2 emission reduction in Chinese heavy industries: Do environmental regulations matter?	Chinese pilot ETS	Industry	DiD
Peng et al. (2021)	The environmental and economic effects of the carbon emissions trading scheme in China: The role of alternative allowance allocation	Chinese pilot ETS	All sectors covered by the carbon price	DiD
Qi et al. (2021)	Environmental and economic effects of China's carbon market pilots: Empirical evidence based on a DID model	Chinese pilot ETS	Whole economy	DiD
Shen et al. (2020)	Does China's carbon emission trading reduce carbon emissions? Evidence from listed firms	Chinese pilot ETS	All sectors covered by the carbon price	DiD
Tang et al. (2021)	The effectiveness and heterogeneity of carbon emissions trading scheme in China	Chinese pilot ETS	All sectors covered by the carbon price	DiD
Wang et al. (2019)	Effect of the Emissions Trading Scheme on CO2 Abatement in China	Chinese pilot ETS	Whole economy	DiD
Wang et al. (2022)	Can China's carbon trading policy help achieve Carbon Neutrality?-A study of policy effects from the Five-sphere Integrated Plan perspective	Chinese pilot ETS	Whole economy	DiD
Wang, Liao et al. (2021)	The impact of foreign direct investment on China's carbon emissions through energy intensity and emissions trading system	Chinese pilot ETS	Whole economy	DiD
Wang, Shi et al. (2021)	The policy effects and influence mechanism of China's carbon emissions trading scheme	Chinese pilot ETS	All sectors covered by the carbon price	DiD
Wen et al. (2020)	Does China's carbon emissions trading scheme really work? A case study of the hubei pilot	Chinese pilot ETS	Industry	DiD
Wen et al. (2021)	Environmental and economic performance of China's ETS pilots: New evidence from an expanded synthetic control method	Chinese pilot ETS	Industry	DiD
Wu et al. (2021)	Examining the Impact and Influencing Channels of Carbon Emission Trading Pilot Markets in China	Chinese pilot ETS	All sectors covered by the carbon price	DiD
Xu (2021)	The Impact and Influencing Path of the Pilot Carbon Emission Trading market–Evidence From China	Chinese pilot ETS	All sectors covered by the carbon price	DiD

Table S5 – *Continued from previous page*

Authors (year)	Title	Scheme	Studied sector	Study design
Yang et al. (2020)	Does China's carbon emission trading policy have an employment double dividend and a Porter effect?	Chinese pilot ETS	All sectors covered by the carbon price	DiD
Yang et al. (2021)	Will China's low-carbon policy balance emission reduction and economic development? Evidence from two provinces	Chinese pilot ETS	All sectors covered by the carbon price	DiD
Yang et al. (2022)	Carbon Emission Trading Scheme, Carbon Emissions Reduction and Spatial Spillover Effects: Quasi-Experimental Evidence From China	Chinese pilot ETS	All sectors covered by the carbon price	DiD
Yi et al. (2020)	Evaluation on the effectiveness of China's pilot carbon market policy	Chinese pilot ETS	All sectors covered by the carbon price	DiD
Zhang & Zhang (2019)	Estimating the impacts of emissions trading scheme on low-carbon development	Chinese pilot ETS	Whole economy	DiD
Zhang, Duan & Deng (2019)	Have China's pilot emissions trading schemes promoted carbon emission reductions?- the evidence from industrial sub-sectors at the provincial level	Chinese pilot ETS	All sectors covered by the carbon price	DiD
Zhang, Duan & Zhang (2019)	Analysis of the Impact of China's Emissions Trading Scheme on Reducing Carbon Emissions	Chinese pilot ETS	All sectors covered by the carbon price	DiD
Zhang, Li, Li & Gao (2020)	Emission reduction effect and carbon market efficiency of carbon emissions trading policy in China	Chinese pilot ETS	Industry	DiD
Zhang, Li, Luo & Gao (2020)	The effect of emission trading policy on carbon emission reduction: Evidence from an integrated study of pilot regions in China	Chinese pilot ETS	Whole economy	DiD
Zhang, Zhang & Yu (2019)	Carbon mitigation effects and potential cost savings from carbon emissions trading in China's regional industry	Chinese pilot ETS	Industry	DiD
Zhang, Zhang et al. (2019)	Sustainable Feasibility of Carbon Trading Policy on Heterogenetic Economic and Industrial Development	Chinese pilot ETS	Whole economy	DiD
Zhang, Zhang et al. (2020)	Has China's Emission Trading System Achieved the Development of a Low-Carbon Economy in High-Emission Industrial Subsectors?	Chinese pilot ETS	All sectors covered by the carbon price	DiD
Best et al. (2020)	Carbon Pricing Efficacy: Cross-Country Evidence	cross-country	Whole economy	control/treatment

Table S5 – *Continued from previous page*

Authors (year)	Title	Scheme	Studied sector	Study design
Ko & Lee (2021)	Carbon pricing and decoupling between greenhouse gas emissions and economic growth: A panel study of 29 European countries, 1996–2014	cross-country	Whole economy	control/treatment
Metcalf & Stock (2020)	The Macroeconomic Impact of Europe's Carbon Taxes	cross-country	All sectors covered by the carbon price	before/after treatment
Rafaty et al. (2021)	Carbon pricing and the elasticity of CO2 emissions	cross-country	All sectors covered by the carbon price	DiD
Bayer & Aklin (2020)	The European Union Emissions Trading System reduced CO2 emissions despite low prices	EU ETS	All sectors covered by the carbon price	DiD
Clo et al. (2017)	Ownership and environmental regulation: Evidence from the European electricity industry	EU ETS	Energy	before/after treatment
Colmer et al. (2022)	Does pricing carbon mitigate climate change? Firm-level evidence from the European Union emissions trading scheme	EU ETS	Industry	DiD
Dechezleprêtre et al. (2018)	The joint impact of the European Union emissions trading system on carbon emissions and economic performance	EU ETS	All sectors covered by the carbon price	DiD
Fageda & Teixido (2022)	Pricing carbon in the aviation sector: Evidence from the European emissions trading system	EU ETS	Transport	DiD
Fernandez et al. (2018)	Institutional Change and Environment: Lessons from the European Emission Trading System	EU ETS	Whole economy	before/after treatment
Gupta et al. (2021)	Causal Impact Of European Union Emission Trading Scheme On Firm Behaviour And Economic Performance: A Study Of German Manufacturing Firms	EU ETS	Industry	DiD
Heiaas (2021)	The EU ETS and Aviation: Evaluating the Effectiveness of the EU Emission Trading System in Reducing Emissions from Air Travel	EU ETS	Transport	DiD
Jaraite & Di Maria (2016)	Did the EU ETS Make a Difference? An Empirical Assessment Using Lithuanian Firm-Level Data	EU ETS	All sectors covered by the carbon price	DiD
Klemetsen et al. (2020)	The impacts of the EU ETS on Norwegian plants' environmental and economic performance	EU ETS	All sectors covered by the carbon price	DiD

Table S5 – *Continued from previous page*

Authors (year)	Title	Scheme	Studied sector	Study design
Petrick & Wagner (2014)	The impact of carbon trading on industry: Evidence from German manufacturing firms	EU ETS	Industry	DiD
Wagner et al. (2014)	The causal effects of the European Union Emissions Trading Scheme: evidence from French manufacturing plants	EU ETS	Industry	DiD
Gugler et al. (2021)	Effectiveness of climate policies: Carbon pricing vs. subsidizing renewables	EU ETS, UK carbon price support	Energy	price elasticity
Elbaum (2021)	The effect of a carbon tax on per capita carbon dioxide emissions: evidence from Finland	Finnish carbon tax	Transport	DiD
Mideksa (2021)	Pricing for a Cooler Planet: An Empirical Analysis of the Effect of Taxing Carbon	Finnish carbon tax	Transport	DiD
Kim & Bae (2022)	Do firms respond differently to the carbon pricing by industrial sector? How and why? A comparison between manufacturing and electricity generation sectors using firm-level panel data in Korea	Korea ETS	Industry	
Hanoteau & Talbot (2019)	Impacts of the Quebec carbon emissions trading scheme on plant-level performance and employment	Quebec ETS	All sectors covered by the carbon price	DiD
Chan & Morrow (2019)	Unintended consequences of cap-and-trade? Evidence from the Regional Greenhouse Gas Initiative	RGGI	All sectors covered by the carbon price	DiD
Murray & Maniloff (2015)	Why have greenhouse emissions in RGGI states declined? An econometric attribution to economic, energy market, and policy factors	RGGI	Energy	DiD
Yan (2021)	The impact of climate policy on fossil fuel consumption: Evidence from the Regional Greenhouse Gas Initiative (RGGI)	RGGI	Energy	DiD
Zhou & Huang (2021)	How regional policies reduce carbon emissions in electricity markets: Fuel switching or emission leakage	RGGI	Energy	DiD
Hamamoto (2021)	Target-setting emissions trading program in saitama prefecture: Impact on CO2 emissions in the first compliance period	Saitama ETS	All sectors covered by the carbon price	DiD

Table S5 – *Continued from previous page*

Authors (year)	Title	Scheme	Studied sector	Study design
Sadayuki & Arimura (2021)	Do regional emission trading schemes lead to carbon leakage within firms? Evidence from Japan	Saitama ETS, Tokyo ETS	All sectors covered by the carbon price	DiD
Yajima et al. (2021)	Energy consumption in transition: Evidence from facility-level data	Saitama ETS, Tokyo ETS	Industry	DiD
Andersson (2019)	Carbon Taxes and CO2 Emissions: Sweden as a Case Study	Swedish carbon tax	Transport	DiD
Runst & Thonipara (2020)	Dosis facit effectum why the size of the carbon tax matters: Evidence from the Swedish residential sector	Swedish carbon tax	Buildings	DiD
Hintermann & Žarković (2021)	A carbon horse race: abatement subsidies vs. permit trading in Switzerland	Swiss ETS	All sectors covered by the carbon price	DiD
Abe & Arimura (2021)	An empirical study of the tokyo emissions trading scheme: An ex post analysis of emissions from university buildings	Tokyo ETS	Buildings	DiD
Arimura & Abe (2021)	The impact of the Tokyo emissions trading scheme on office buildings: what factor contributed to the emission reduction?	Tokyo ETS	Industry	DiD
Abrell et al. (2022)	How effective is carbon pricing?—A machine learning approach to policy evaluation	UK carbon price support	Energy	before/after treatment
Gugler et al. (2022)	Carbon pricing and emissions: Causal effects of Britain's carbon tax	UK carbon price support	Energy	before/after treatment
Leroutier (2022)	Carbon pricing and power sector decarbonization: Evidence from the UK	UK carbon price support	Energy	DiD

Outlier detection

We assess the influence of single studies on the estimated average effect, using the multilevel random effects model. We calculate the Cook's distance and DFBETAS. The values are depicted in Fig. S1. The figure shows that the influence of three studies (4685975_1, 4685975_2, 5196237_2) is considerably higher than for the rest of the sample. All three of these studies assess the effect of the policy on emissions from the burning of coal. These effects likely result from fuel switching without capturing the overall emission effect. We therefore decided to exclude a total of 13 effect sizes from five studies, which focus on emissions from coal. Table S1 shows that the average treatment effect including the outliers would result in a 2 percentage points larger emission reduction estimate.

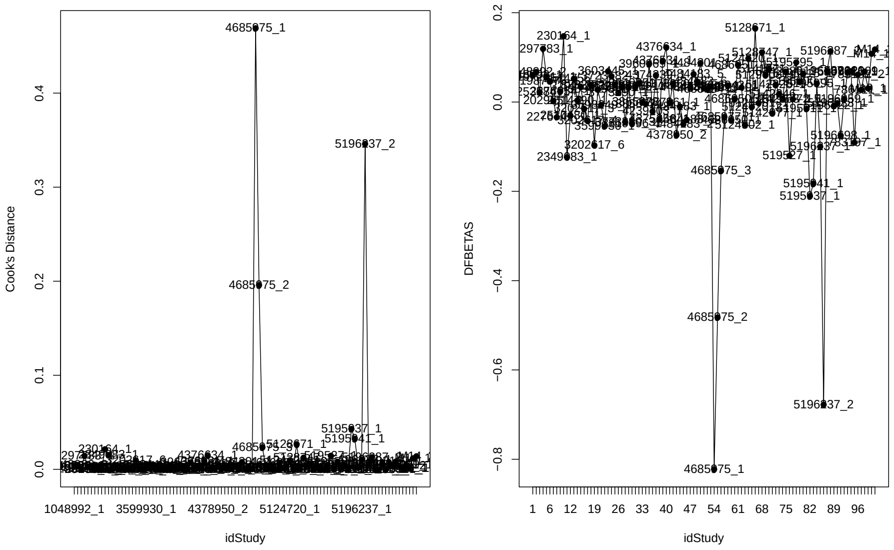

Figure S1: **Assessment of the influence of single studies on the average treatment effect:** Cook's distance (left) and DFBETAS (right) both identify an undue influence of the same three studies.

Table S6: Availability of ex-post evaluations of emission reductions by policy

Scheme name	Type of scheme	Year of implementation	State of research
Alberta TIER	ETS	2007	research gap
Argentina carbon tax	Carbon tax	2018	research gap
Austria ETS	ETS	2022	research gap
BC GGIRCA	ETS	2016	research gap
BC carbon tax	Carbon tax	2008	covered
Beijing pilot ETS	ETS	2013	covered
California CaT	ETS	2012	covered
Canada federal OBPS	ETS	2019	research gap
Canada federal fuel charge	Carbon tax	2019	research gap
Chile carbon tax	Carbon tax	2017	research gap
China national ETS	ETS	2021	research gap
Chongqing pilot ETS	ETS	2014	covered
Colombia carbon tax	Carbon tax	2017	research gap
Denmark carbon tax	Carbon tax	1992	research gap
EU ETS	ETS	2005	covered
Estonia carbon tax	Carbon tax	2000	research gap
Finland carbon tax	Carbon tax	1990	covered
France carbon tax	Carbon tax	2014	research gap
Fujian pilot ETS	ETS	2016	covered
Germany ETS	ETS	2021	research gap
Guangdong pilot ETS	ETS	2013	covered
Hubei pilot ETS	ETS	2014	covered
Iceland carbon tax	Carbon tax	2010	research gap
Ireland carbon tax	Carbon tax	2010	research gap
Japan carbon tax	Carbon tax	2012	research gap
Kazakhstan ETS	ETS	2013	research gap
Korea ETS	ETS	2015	covered
Latvia carbon tax	Carbon tax	2004	research gap
Liechtenstein carbon tax	Carbon tax	2008	research gap
Luxembourg carbon tax	Carbon tax	2021	research gap
Massachusetts ETS	ETS	2018	research gap
Mexico carbon tax	Carbon tax	2014	research gap
Mexico pilot ETS	ETS	2020	research gap
Montenegro ETS	ETS	2022	research gap
Netherlands carbon tax	Carbon tax	2021	research gap
New Brunswick ETS	ETS	2021	research gap
New Brunswick carbon tax	Carbon tax	2020	research gap
New Zealand ETS	ETS	2008	research gap
Newfoundland and Labrador PSS	ETS	2019	research gap

Table S6 – *Continued from previous page*

Scheme name	Type of scheme	Year of implementation	State of research
Newfoundland and Labrador carbon tax	Carbon tax	2019	research gap
Northwest Territories carbon tax	Carbon tax	2019	research gap
Norway carbon tax	Carbon tax	1991	research gap
Nova Scotia CaT	ETS	2019	research gap
Ontario EPS	ETS	2022	research gap
Oregon ETS	ETS	2021	research gap
Poland carbon tax	Carbon tax	1990	research gap
Portugal carbon tax	Carbon tax	2015	research gap
Prince Edward Island carbon tax	Carbon tax	2019	research gap
Quebec CaT	ETS	2013	covered
Queretaro carbon tax	Carbon tax	2022	research gap
RGGI	ETS	2009	covered
Saitama ETS	ETS	2011	covered
Saskatchewan OBPS	ETS	2019	research gap
Shanghai pilot ETS	ETS	2013	covered
Shenzhen pilot ETS	ETS	2013	covered
Singapore carbon tax	Carbon tax	2019	research gap
South Africa carbon tax	Carbon tax	2019	research gap
Spain carbon tax	Carbon tax	2014	research gap
State of Mexico carbon tax	Carbon tax	2022	research gap
Sweden carbon tax	Carbon tax	1991	covered
Switzerland ETS	ETS	2008	covered
Switzerland carbon tax	Carbon tax	2008	research gap
Tianjin pilot ETS	ETS	2013	covered
Tokyo CaT	ETS	2010	covered
UK Carbon Price Support	Carbon tax	2013	covered
UK ETS	ETS	2021	research gap
Ukraine carbon tax	Carbon tax	2011	research gap
Uruguay CO2 tax	Carbon tax	2022	research gap
Washington CCA	ETS	2023	research gap
Yucatan carbon tax	Carbon tax	2022	research gap
Zacatecas carbon tax	Carbon tax	2017	research gap

Note: The table shows for which carbon pricing schemes we identified ex-post evidence estimating the emission reduction caused by the policy or where research gaps remain. The information on the name, type, and year of implementation of each of the schemes is based on the information provided by the World Bank's Carbon Pricing Dashboard (status 2023). *State of research* indicates whether we identified relevant ex-post assessments (*covered*) or not (*research gap*).

Codebook

We capture two levels of information: study level and effect level information. We do the first by using the NACSOS software[1] which automatically records study level information concerning authors, publication year, publication name and publication type, where available from the searched databases. We then use the NACSOS software for relevance coding on the title-abstract level. Our search strategy is described in detail in our protocol.

This document describes the coding at the study level and the effect level done in Excel. For studies which appeared relevant on the title-abstract level we first record whether the study fulfils our inclusion criteria after reading the full text. At the study level we further assess the risk of bias, informed by the ROBINS-I framework[2]. We amend the ROBINS-I assessment criteria in order to be applicable to our set of non-randomised observational studies on the impact of an intervention at the aggregated level (e.g. country-level, sector-level). The criteria for our risk of bias assessment are described below. We capture the relevant information on the carbon pricing schemes from the primary studies. Further information on the schemes can be added later from other sources.

Study level information

Field Name	Explanation	Choices or Examples
Include	The inclusion/exclusion criteria are specified in the protocol.	*Options*<br>include<br>exclude
Exclusion reason	If you exclude the article, please specify why you exclude it.	*Options*<br>No relevant effects,<br>Effect information incomplete/missing,<br>Other
Exclusion reason description	Please describe your decision for exclusion further.<br><br>You have put some thoughts into this so it is the right time to verbalise them. If someone “challenges” your decision later, it will be very helpful to have these thoughts recorded.<br><br>Especially if you had to exclude the study because of missing or incomplete information, all pieces of information provided here will be	e.g.<br>“The study only analyses leakage effects of the policy intervention, but does not provide any estimates on the development of carbon emissions within the territory of its application.",<br>“The study only provides descriptive information on the carbon emission path but does not estimate any causal effects regarding the implementation of the intervention."

[1] More information on the NACSOS software can be found at https://github.com/mcallaghan/nacsos.
[2] More information on the ROBINS-I framework can be found at http://www.riskofbias.info.

<table>
<tr><td></td><td>very helpful in the subsequent process of trying to recapture the missing information. Also provide as accurately as possible information about in which table or paragraph and on which page you would expect the missing information.</td><td></td></tr>
<tr><td colspan="3">The subsequent labels cover the risk of bias assessment. The assessment is inspired by the ROBINS-I tool which is based on the Cochrane risk of bias tool for randomized trials. All studies in our sample are non-randomized intervention studies working with real-world policies and real-world data. As such, study results are at risk not to report the true effect of the intervention on the outcome variable but to report biased estimates influenced by confounding factors, selective reporting, or other biases. The subsequent questions assess how severe this risk is. The benchmark should always be an idealised study, which can perfectly single out the effect of the intervention on the dependent variable. You can select from five different answers: Yes, Probably Yes, Probably No, No, No Information. Not applicable can be selected for some questions if the question does not apply to the study design. The answers should be selected according to the following judgement.
Yes: Critical risk of bias (the study design is too problematic in this domain to provide any useful evidence on the effects of intervention);
Probably Yes: Serious risk of bias (the study has some important problems in this domain);
Probably No: Moderate risk of bias (the study is sound for a non-randomized study with regard to this domain but cannot be considered comparable to a well-performed randomized trial);
No: Low risk of bias (the study is comparable to a well-performed randomized trial with regard to this domain);
No information: There is no information on which to base a judgement about risk of bias for this domain.</td></tr>
<tr><td>RoB – selection of treated study objects</td><td>Is there a risk of bias in the selection of treated study objects?

The treatment in this context is independent of the research design and subject to political decisions of the jurisdictions. The study should cover all treated study objects or a representative sample.

We judge the risk of bias with respect to the country-sector-fuel analysed by the authors. So if the study e.g. focuses on the energy sector, this does not lead to an increased risk of bias.</td><td>*Options*
Yes,
Probably Yes,
Probably No,
No,
No Information</td></tr>
<tr><td>RoB – selection of control group</td><td>Is there a risk of bias in the selection of the control group (if relevant for the study design)?
In the idealized setting the control group would perfectly mimic the development of the emissions in the treatment group in absence of the carbon pricing policy.
Risk of bias can be reduced if the control group is chosen based on regional, institutional, cultural, or economic arguments (e.g., comparing neighbouring countries or regions of the same country). It should be reasonably</td><td>Yes,
Probably Yes,
Probably No,
No,
No Information,
Not applicable</td></tr>
</table>

	outlined how the control group compares with the treatment group in decisive factors. In particular pre-intervention GHG pathways and other descriptive statistics should be compared. Risk of bias can be further reduced through the application of study designs which increase the comparability of the control group with the treatment group, like matching or synthetic controls.	
RoB – selection description	Describe or use quotes from the paper which illustrate how the authors deal with the risk of bias from the selection of treatment and control group.	
RoB – confounding factors	<u>Is there a risk of confounding factors, which are not appropriately controlled for?</u><br>*The risk can be reduced if the estimation procedure reasonably controls for confounding factors. Such confounding factors could either be differences in characteristics between the treatment and control group which could, independent of the treatment, have an effect on the emissions (dependent variable), like initial GHG emissions, environmental awareness, country factors like GDP, governance or other environmental regulation/policy. Other confounding factors could be other events, like the global financial crisis or the implementation of other relevant policies, happening during the study period, which could have a significant effect on the emissions.*<br>*The selection of control variables (and exclusion of others) should be reasonably justified and discussed with reference to the relevant literature as well as with robustness analyses of the findings with respect to the inclusion/exclusion of critical control variables.*<br><br><u>If instrumental variables (IV) are used, is there a risk of bias due to an invalid IV?</u><br>*The validity of the IV should be reasonably justified. In particular the relevance condition and the exclusion restriction should hold. For the relevance condition, tests should be provided,*	Yes,<br>Probably Yes,<br>Probably No,<br>No,<br>No Information,<br>Not applicable

	which show that the instrument is a relevant predictor for the observed variation in the explanatory variable (the policy intervention). For the exclusion restriction it should be credibly justified that the instrument only affects the emissions (dependent variable) through the explanatory variable (is it credible that the (changes in) greenhouse gas emissions/fuel consumption were only affected by the instrument through the variation in carbon pricing?).<br><br><u>If a regression discontinuity in time design is used, is there a risk of bias due to the identifying assumptions underlying the design?</u><br>*For the justification of the identifying assumptions, in particular the political and economic context should be discussed as well as (graphical) evidence provided for the smoothness of trends in observed control variables around the cutoff. Robustness checks with respect to a varying bandwidth around the cutoff as well as with respect to the inclusion of higher order polynomials in the outcome (emissions/fuel consumption) trends before and after the cutoff further reduce the risk of bias.*	
RoB – confounding factors Description	Describe or use quotes from the paper which illustrate how the authors deal with the risk of bias caused by confounding factors.	
RoB – other	Are there any other biases in the study? If the validity of the study is restricted by any other factors, please describe the present biases in the next label.<br>*One possible bias could arise in price elasticity studies from the endogeneity of carbon prices and carbon emissions. This can particularly be the case if prices are formed on markets and the emission levels of single study objects have a significant influence on the price.*	Yes,<br>Probably Yes,<br>Probably No,<br>No
RoB – other description	Please describe any other present biases here and/or paste in quotes from the paper.	

Effect level information

For included studies we will subsequently record information at the effect size level, i.e. the estimated effects of carbon pricing on one of the outcome variables of interest. The risk of bias assessment does not influence the inclusion/exclusion of the study and all further information shall be captured regardless of the risk of bias assessment.

Each study may report multiple estimates of the effect. This can be due to different model specifications, different control groups, different periods, separately calculated effects for different sectors, etc. You are asked to capture all the *relevant* effect sizes and corresponding information reported by the study, this includes effects reported in supplementary information such as the appendix. Obviously, model specifications which do not contain relevant effect sizes (e.g. if the carbon pricing variable was excluded from a model specification) can and should not be captured.

Field Name	Explanation	Choices or Examples
Effect size type	Introduction (treatment) effects specify the effect of the presence of the policy compared to its absence. This would usually be done using a dummy variable.<br>A price elasticity is estimated using the carbon price level as an explanatory variable for the emission level.	Introduction effect<br>Price elasticity
Effect number	Please assign numbers to the effects. Start with the first reported effect size in the main text. Do not use the numbers assigned to columns in tables of results as these may be ambiguous (there may be several tables of results).	1, 2, 3,…
Location of the information	Please note down where the effect size (and related uncertainty measures etc.) was found. Was it captured in a table, chart or a text paragraph?<br><br>If available, please use the page numbers of the article, not the pdf. If no other page numbers are available, use the page numbers of the pdf.<br><br>Please keep in mind, that it might be necessary to go back to individual studies at some point in a later project phase. If any additional information on the location of the values is	page 683; table 4; column 1,<br>page 89; table 4; column 5 and page 90 second paragraph,<br>page 9; paragraph 16<br>appendix B; page 5; table A.3; column 4;

Field Name	Explanation	Choices or Examples
	needed to find the information again, please capture that here.	
Study design type	Choose one or multiple of the options.<br><br>Please contact us, if you discover a study design which does not fit the predefined options, or if you are unsure which category to choose.	DiD,<br>DiDiD,<br>Matching,<br>synthetic control,<br>2SLS,<br>Regression discontinuity/structural break,<br>Panel without fixed effects,<br>Panel with fixed effects,<br>time series OLS (pure time series),<br>OLS (pure cross section),<br>other
Dependent variable co2/GHG	How is the dependent variable defined?<br>If the dependent variable originally is not in CO2 emissions or CO2 equivalents but can be recalculated with information provided by the authors, perform the calculation into CO2 equivalents on your own and select Greenhouse gases.	CO2,<br>Greenhouse gases (in CO2 equivalents)
Dependent variable total/capita	How is the dependent variable defined?	Total,<br>Per capita
Dependent variable levels/growth rates	How is the dependent variable defined?	Levels,<br>Growth rates
Log-transformations of dependent and independent variables	Capture whether the dependent and/or independent variables are transformed to log values.<br><br>e.g., choose log-level if the dependent variable is in logs and the independent variable is in levels.	Level-level,<br>Log-Level,<br>Log-Log,<br>Level-Log,<br>relative change in percent/100

Field Name	Explanation	Choices or Examples
Larger risk-of-bias dummy	Here we are interested in the risk of bias of individual estimates relative to the risk of bias assessment on the study level.<br><br>Does the estimate have a larger risk of bias then the best estimates (i.e. the estimates with the lowest risk of bias, included in the study).<br><br>E.g., this can be estimates which have been estimated without the control variables deemed necessary by the authors or without fixed effects etc. Some robustness checks may also fall into this category.	Yes/No
Larger risk-of-bias - Explanation	If you assessed the estimated effect size to have a larger risk of bias, please explain what causes the higher risk of bias.	e.g.<br>“The effect size was estimated without fixed effects.",<br>“The effect size was estimated leaving out the control variables for GDP."
Complete effect size information	For the meta regression we need information on the effect size as well as complete information on at least one uncertainty measure.	yes/no
Effect size – statistical estimate	Capture the statistical estimate of the effect of the explanatory variable (introduction/existence of carbon pricing/carbon price) on the outcome variable (emissions etc.) as given by the authors.	
Effect size – statistical estimate standardized	We want to standardize effect sizes to make them comparable across studies. If the effect sizes in a study require recalculations to fit the required format, carry out the necessary recalculations and capture the standardized effect size.<br><br>If the effect is given in percent, divide it by 100.<br><br>If the effect size is given in CO2 (equivalent) capture it in tons.	

Field Name	Explanation	Choices or Examples
Effect size - direction	Capture the direction of the effect carbon pricing had on the outcome variable.<br><br>If the effect suggests that carbon pricing decreased the outcome variable relative to a baseline/control scenario choose “decrease”. Otherwise choose “increase”.	increase,<br>decrease
Cumulative-periodic effect size	Capture in which form the effect size is provided. If a cumulative emission reduction is provided for the full time period from the time of introduction until the end of the observation period, select "cumulative". If the reduction is reported as the reduction in each observation point (e.g., for annual observations, the annual reduction), select “periodic”.<br>If the reduction in each period is estimated choose “periodic”.	Cumulative,<br>Periodic,<br>other
Uncertainty measure	Capture the uncertainty measure provided for the effect size. Fill in all provided uncertainty measures.<br><br>If only a confidence interval is provided capture the confidence interval in the following form: confidence interval; lower bound; upper bound.	standard error<br>t-statistic<br>p-value<br>confidence interval (upper and lower bound)
Uncertainty measure standardized	If the statistical estimate had to be recalculated for standardization, the uncertainty measure needs to be recalculated accordingly.	
Sample Size	Capture the observed entities/individuals with as much detail as possible (i.e. if all three options are given, record all).<br><br>**Total** – Full sample size (for synthetic control estimations, capture the treatment group and the full donor pool)<br>**Treatment** – Capture sample size of entities with carbon pricing<br>**Control** – Capture the sample size of entities without carbon pricing.	In a clinical study, this would be the number of participants in the trial.

Field Name	Explanation	Choices or Examples
Number of observations	If panel data is analysed the number of observations in a regression differs from the sample size of the study.	
Significance level	If the study indicates the level of significance at which the effect size is significant, capture the highest level of indicated significance.<br><br>Often this indication of significance is provided by asterisks/stars in the results table or it is mentioned in the text.	<0.90,<br>0.90,<br>0.95,<br>0.99,<br>>0.99,<br>Insignificant,<br>not reported,
Main specification	Choose 'main specification' to indicate if the authors describe this estimate as their main specification.	main specification
Mean carbon price	Capture the mean carbon price level of the treated countries as specified in the study. This is manly important, if the study estimates a price elasticity.<br><br>For price semi-elasticities the carbon price entered here should correspond to the semi-elasticity recorded in the "Effect size – statistical estimate" field such that captured semi-elasticity can be transformed to an introduction effect by multiplying it with the mean carbon price.<br><br>Only consider observation periods where a carbon price was introduced i.e. the average carbon price between the introduction date of the carbon tax and the end of the treatment period.	
Mean carbon price - currency	Capture the currency of the explanatory variable. If you do not indicate otherwise we assume that prices are current prices.<br><br>It is preferrable to capture the mean price level in constant prices, if given in the paper.	Euro<br>US dollar<br>Canadian dollar<br>Chinese Yuan<br>New Zealand dollar<br>Norwegian krone

Field Name	Explanation	Choices or Examples
	If the carbon price is not given per ton of CO_2-equivalent, specify the currency and the quantity of fuel the carbon price is referring to (see example).	Swedish krona<br>UK pound sterling<br><br><br>Canadian cents per litre of gasoline
Mean carbon price – currency base year	Please specify the base year if a paper reports constant prices.	
Mean carbon price - **standardized**	Capture the mean carbon price in currency unit per ton of CO2.<br><br>For price semi-elasticities the carbon price entered here should correspond to the semi-elasticity recorded in the "Effect size – statistical estimate **standardized**" field such that captured semi-elasticity can be transformed to an introduction effect by multiplying it with the mean carbon price standardized.	
Mean carbon price – currency **standardized**	As specified above but for the standardized mean carbon price.	
Mean carbon price – currency base year **standardized**	As specified above but for the standardized mean carbon price.	
Explanatory variable - currency	Capture the currency of the explanatory variable. If you do not indicate otherwise we assume that prices are current prices. This is manly important, if the study estimates a price elasticity.	Euro<br>US dollar<br>Canadian dollar<br>Chinese Yuan<br>New Zealand dollar<br>Norwegian krone<br>Swedish krona<br>UK pound sterling
Explanatory variable – currency base year	Please specify the base year, if papers use constant prices.	
Introduction date	Capture the year when the carbon price analysed in the study was introduced.	1999,<br>2005,<br>01.07.2015

Field Name	Explanation	Choices or Examples
	If the carbon price was not introduced on January 1, capture the full introduction date in the form DD.MM.YYYY	
Start date	We would like to capture the period which the calculation of the effect is based on.<br><br>Capture the start of the observation period.<br><br>If the observation period does not start on January 1, use the format DD.MM.YYYY	*e.g.*<br>2005,<br>01.03.2003<br><br>In a synthetic control study, capture the first year of the period on which the calculation of the weights is based on.
End date	Fill in the last year of the treatment period here. If the observation period does not end on December 31, use the format DD.MM.YYYY	2017,<br>01.12.2018
Time aggregation level	Capture the level of aggregation for the emission data on a time dimension.<br><br>Choose one of the options.	Larger than year,<br>Year,<br>Quarter,<br>Month,<br>Lower than a month
Entity aggregation	Capture the entity aggregation level at which the data is being analysed.<br><br>Choose one option. If, for example, the observations are aggregated by sector and country, choose both.	block,<br>country,<br>state/region,<br>city,<br>firm,<br>plant,<br>sector
Mean emissions	EITHER 1.) Capture the mean annual emissions in tonnes of CO_2 (or CO_2 equivalents), if provided or if they can be calculated. Then standardize the effect size to match the emission reduction in tonnes and enter it in the field effectSize.statisticalEstimate.standardized OR 2.) Capture in the same unit as the effectSize.statisticalEstimate such that the ratio between effectSize.statisticalEstimate/mean(emissions) can be interpreted as the percentage change in emissions.	The mean emissions should have the same base, as the effect, e.g., if the effect is based on a per capita calculation, capture the mean emissions per capita.<br><br>If you have to perform recalculations: 1 year = 365 days

Field Name	Explanation	Choices or Examples
	Preference order for the recording of mean emissions:<br>(1) mean annual counterfactual emissions during the treatment period<br>(2) mean annual emissions of the treatment group during the treatment period<br>(3) mean annual emissions of the full sample during the treatment period<br>(4) mean annual emissions of the full sample over the full observation period<br><br>Specify any thoughts you put into your selection in the notes.	
Geographic location - treatment group	Capture the geographic location for which emission data was analysed. Here we do not look for the scope of the intervention. For the effect of the EU ETS on emission from the German electricity sector the value will be *Germany.*<br>Note: Try and stick to the format city/state;country/block.<br>In case that there is more than one entity analysed connect them with a comma.<br>city/state;country/block,city/state;country/bloc k<br>If a block of locations was analysed and there exists an unambiguous name, put the name.<br><br>Remove all white spaces. If country names require a whitespace, replace it with a lower bar.<br><br>As ambiguity in spelling names is common, please consider the following partial list:<br>British_Columbia<br>Be**ij**ing<br>China<br>Hubei<br>New_Z**ea**land<br>USA	*e.g.*<br>city/state;country/block<br>Berlin;Germany,<br>France,<br>Pennsylvania;USA,<br>Shenzhen;China.<br>British_Columbia;Canada<br><br>EU-25,<br>EU-27,<br>China_pilots (if **all** China pilot jurisdictions are included),<br><br>Regex:<br>\S+

Field Name	Explanation	Choices or Examples
Multiple interventions	Capture if a single carbon pricing intervention was studied or whether multiple carbon pricing interventions are studied. (e.g. does the study analyse the effect of the EU ETS only or does the dataset contain multiple jurisdictions with multiple carbon pricing policies.)<br><br>Choose one of the options.	single,<br>multiple
Emission sector	Capture the emission sector which the analysis is performed on.<br><br>Choose one or multiple options.<br><br>Note: if sectors are not specified but it is indicated that all sectors covered by an intervention are analysed choose *all covered sectors.*	*Options (sector definition of the IPCC)*<br>Energy,<br>Industry,<br>Transport,<br>Buildings,<br>AFOLU,<br>International Aviation and Shipping,<br>all covered sectors,<br>economy,
Fuel type	If only a specific fuel type is analysed choose the type accordingly. Leave unanswered otherwise.	Coal,<br>Natural gas,<br>Petrol,<br>Gasoline,<br>Diesel
Sub-category	If only a subset of the sector or fuel is analysed, please specify the sector/fuel here.	Residential buildings only
Used data	Capture the data source used for the dependent variable. In particular, which database was used to extract (or calculate) the carbon/GHG emissions.<br><br>Note: *Try* and stick to the format: dataset provider;dataset name;version/publication year	e.g.<br>International Energy Agency;CO2 Emissions from Fuel Combustion; 2019,<br>EU;European Union Transaction Log
Notes	Any notes.	
Coding completed	The date when the coding was completed. Stick to the format:<br>DD.MM.YYYY	*15.11.2021*
